# Adenovirus-Mediated *FasL* Minigene Transfer Endows Transduced Cells with Killer Potential

**DOI:** 10.3390/ijms21176011

**Published:** 2020-08-20

**Authors:** Madalina Dumitrescu, Violeta Georgeta Trusca, Lorand Savu, Ioana Georgeta Stancu, Attila Cristian Ratiu, Maya Simionescu, Anca Violeta Gafencu

**Affiliations:** 1 Gene Regulation and Molecular Therapies Laboratory, Institute of Cellular Biology and Pathology “N. Simionescu”, 8, B.P. Hasdeu Street, 050568 Bucharest, Romania; madalina.dumitrescu@icbp.ro (M.D.); violeta.trusca@icbp.ro (V.G.T.); maya.simionescu@icbp.ro (M.S.); 2 Molecular Biology Department, Genetic Lab, 9 Căpitan Nicolae Drossu Street, 012071 Bucharest, Romania; lorand_savu@yahoo.com (L.S.); stancu.ioanageorgeta@yahoo.com (I.G.S.); attilaratiu@yahoo.com (A.C.R.)

**Keywords:** FasL, minigene, cDNA, adenovirus, apoptosis

## Abstract

Fas ligand (First apoptosis signal ligand, FasL, also known as CD95L) is the common executioner of apoptosis within the tumor necrosis factor (TNF) superfamily. We aimed to induce functional FasL expression in transduced cells using an adenovirus vector, which has the advantage of strong and transient induction of the gene included in the adenoviral genome. Here, we report that the adenovirus carrying a truncated *FasL* gene, named *FasL* minigene, encoding the full-length FasL protein (Ad-gFasL) is more efficient than the adenovirus carrying FasL cDNA (Ad-cFasL) in the induction of FasL expression in transduced cells. *FasL* minigene (2887 bp) lacking the second intron and a part of the 3′-UTR was created to reduce the gene length due to the size limitation of the adenoviral genome. The results show that, in transduced hepatocytes, strong expression of mRNA FasL appeared after 10 h for Ad-gFasL, while for Ad-cFasL, a faint expression appeared after 16 h. For Ad-gFasL, the protein expression was noticed starting with 0.5 transfection units (TU)/cell, while for Ad-cFasL, it could not be revealed. FasL-expressing endothelial cells induced apoptosis of A20 cells in co-culture experiments. FasL-expressing cells may be exploitable in various autoimmune diseases such as graft-versus-host disease, chronic colitis, and type I diabetes.

## 1. Introduction

Fas ligand (FasL, CD95L) is a type-II transmembrane protein of approximately 40 kDa acting as an executioner of apoptosis within the tumor necrosis factor (TNF) superfamily. FasL activates the caspase cascade through Fas receptor trimerization in target cells. The presence of FasL on the cell surface of the CD8+ cytotoxic T lymphocytes and natural killer cells confers death effector properties [1,2]. FasL is also involved in T-cell development and physiological situations of immunotolerance and immune privilege [3,4,5,6]. Numerous studies have attempted to harness this physiological mechanism of immune regulation for therapeutic purposes, with an unequivocal demonstration of the efficacy of FasL to selectively eliminate activated immune cells, primarily in autoimmune diseases. For example, the FasL protein has been used to restrain inflammatory insulitis in models of autoimmune diabetes [7] and to induce immune privilege in grafted tissues and organs, such as pancreatic islet grafts [8,9]. Therapeutic approaches based on FasL-mediated apoptosis have been designed over the last two decades, following the demonstration that co-transplantation of myoblasts engineered to express FasL protects islet allografts from rejection [8]. Likewise, antigen-presenting cells engineered to express FasL were efficient in the elimination of infiltrating T cells and in prevention of the development of autoimmune attack in numerous epithelial tissues without detectable liver toxicity [10]. Induction of immune privilege has been also achieved using a short-lived FasL protein [11], attributing the protective effects to apoptotic signals and underlying the efficacy of transient FasL-based interventions. Furthermore, a series of studies showed that T regulatory cells decorated with FasL on their surface are effective immunomodulators that alleviate graft-versus-host disease, arrest progression of autoimmune insulitis in non-obese diabetic (NOD) mice, and ameliorate chronic colitis [7,12,13,14,15]. Local administration of FasL was demonstrated to be effective for oral malignant melanoma and osteosarcoma [16,17]. The short lifetime of this molecule is primarily caused by cleavage of the transmembrane FasL with metalloproteinases [4]. Although effective in suppression of immune activity, thus causing immune privilege, short-term therapeutic activity of proteins encoding FasL is not always sufficient in the induction of transplant tolerance and abrogation of autoimmune disorders, which are frequently broken by incidental infections and relapse of autoimmunity [18].

Conceptually, a more sustained expression of the FasL protein may be of superior therapeutic efficacy in some situations, though the high toxicity of this trigger of apoptosis warrants against indefinite expression to restrict any pathology in vivo. Therefore, we conceived that transient expression of a FasL molecule in therapeutic cells may award an advantage in the targeted delivery of apoptotic signals by prolonged yet transient expression of this therapeutic molecule. We aimed to obtain transient expression of FasL on the cell surface without incorporation of the gene in the cell genome, which is best achieved by adenoviral vectors. The major advantage of adenoviral transduction is the lack of a definitive integration of the plasmid into the genome, as compared to other viral methods such as lentiviral and retroviral that induce a permanent expression through genetic integration. An additional advantage is the high yield of the protein expression induced by adenoviruses, as compared to other direct transfection methods, such as lipofection or electroporation. 

Adenoviruses are the most commonly used vectors for gene transfer. The adenoviral genome is represented by the linear double-stranded DNA that encodes for about 30–40 genes. The adenoviral transcription units usually encode for two or more alternatively spliced mRNAs [19]. Due to the compact genome, the regulatory events related to RNA processing are very important for the lytic lifecycle of the adenovirus. The adenoviral genes contain fewer and shorter introns compared to cellular genes. Most viral mRNAs are matured by splicing of one to three introns [20]. The major obstacle for adenoviral gene transfer is the size limitation of the adenoviral genome. The size of the FasL gene (7805 bp excluding promoter) is larger than the maximal acceptable size of the insert in the AdEasy system (5.0kb with pAdEasy1 and 7.7kb with pAdEasy2) [8]. Thus, we truncated the FasL gene and obtained a *FasL* minigene (2887 bp) by exclusion of the large second intron (4102 bp) and part of the 3′-UTR for expression under the control of the CMV promoter. Our data demonstrate the effective expression of a functional FasL molecule by an adenoviral vector on the surface of transduced cells.

## 2. Results

### 2.1. FasL Minigene and FasL cDNA Carrying Adenoviruses

**Figure 1:**
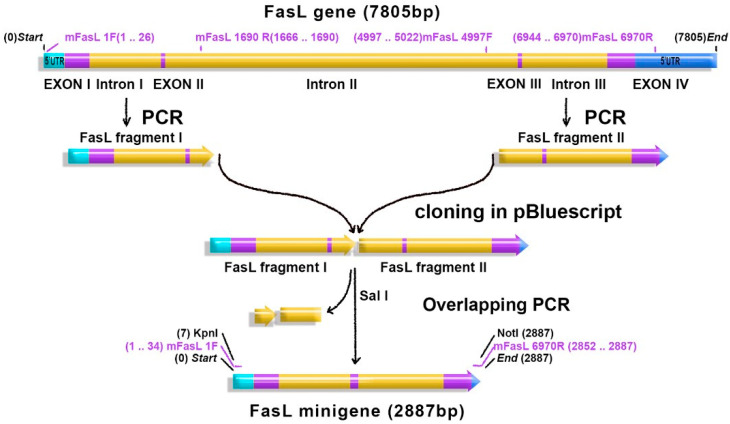
Fas ligand (FasL) minigene construction: the *FasL* murine gene (7805 bp) consists of four exons (purple) and three introns (yellow). Untranslated regions (UTR—blue) flank the introns and the exons. FasL 5′ fragment (FasL fragment I) and FasL 3′ fragment (FasL fragment II) were amplified by PCR and successively cloned in pBluecript. The remaining fragments of the intron II were removed by overlapping PCR, and exons II and III (purple) were linked in frame. The obtained minigene (2887 bp) was cloned in the pAdTrack-CMV adenoviral vector in the KpnI/NotI restriction site.

The minigene comprises the first 6970 bp of the murine FasL gene except the second intron (4102 bp), as illustrated in Figure 1. The minigene was obtained by fusing the 5′ and the 3′-fragments of the gene when the second and the third exons were linked in frame. *FasL* minigene (2887 bp) as well as FasL cDNA (from 170–890 bp in the RNA sequence) were cloned into the pAdTrack-CMV adenoviral vector. The plasmids were evaluated by digestion with restriction enzymes (Figure S1) and were sequenced. The sequence showed that the coding part of the minigene as well as cDNA represents a perfect match with the sequence deposited in the GenBank (No. U58995.1) for BALB/c mice [21]. Regarding the noncoding part of the gene, the sequence contains a substitution in the first intron (649 C→T) and a substitution in the third intron (2054 T→C), as compared with the FasL gene of C57BL/6 mice (GenBank NC_000067.6).

Two PacI restriction sites found in the second intron and in the 3′UTR were also removed by this truncation. The transgene should not have any PacI restriction site since the recombinant plasmid is cleaved with a PacI restriction enzyme to ease transfection of AD293 packaging cells. The adenovirus carrying the *FasL* minigene (Ad-gFasL) and adenovirus carrying FasL cDNA (Ad-cFasL) were similarly prepared by packaging and amplification in AD293 cells stably transfected with a dominant-negative isoform of Fas-associated death domain (FADD) (to confer resistance to FasL-induced apoptosis). In addition, a caspase inhibitor was added to the medium to ensure inhibition of apoptosis of the packaging cells. Adenoviruses were purified from the cell lysates and from the culture media. The purified adenoviruses were titrated in the same time. 

### 2.2. FasL Expression in Transduced Cells

**Figure 2:**
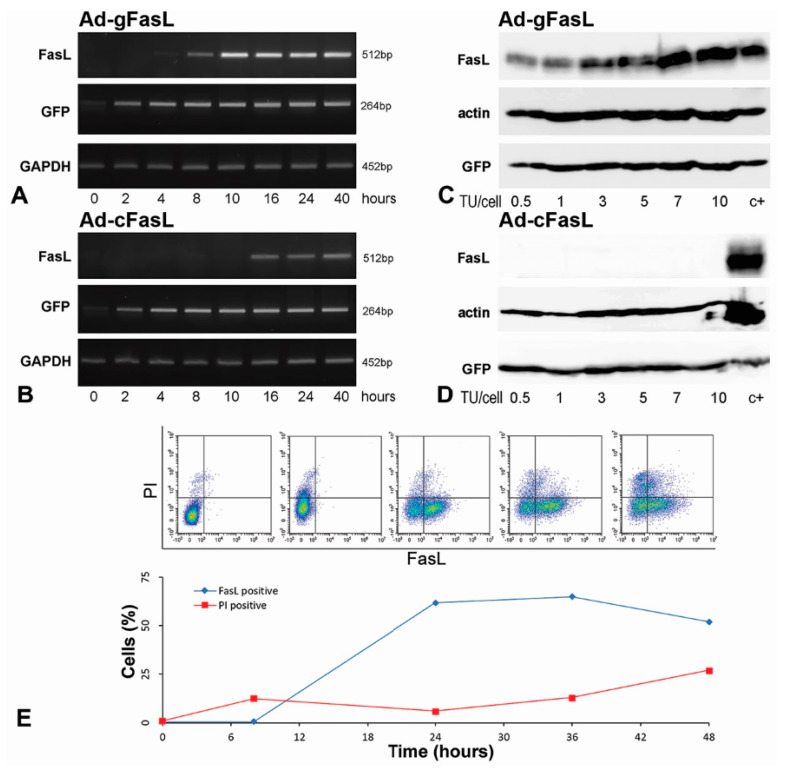
Kinetics of FasL expression in transduced cells: (**A**,**B**) Hepa 1–6 hepatocytes were transduced with 5 transfection units (TU)/cell adenovirus carrying (**A**) *FasL* minigene (Ad-gFasL) and (**B**) cDNA (Ad-cFasL). FasL expression was assessed as a function of time after transduction by RT-PCR. Cells infected with Ad-gFasL displayed FasL expression after 8 h, with significant increased FasL level after 10 h (**A**). The expression of FasL in cells transduced with Ad-cFasL was very low, with delayed onset at 16 h (**B**). In parallel, expression of mRNA encoding GFP and GAPDH were followed. (**C**,**D**) Western blots performed at 40 h after transfection with Ad-gFasL (**C**) and Ad-cFasL (**D**) showed FasL protein expression commensurate with mRNA levels and stable GFP expression under all experimental conditions. Hepa 1–6 cells transfected with the pAdTrack-*FasL* minigene were used as a positive control (c+) in (**C**,**D**). The timecourse of the FasL expression on the membrane of transduced Hepa 1–6 cells was also determined by flow cytometry (**E**). The upper graphs (presented in **E**) represent the evolution in time of the cell population expressing FasL. Quantification of the flow cytometry experiments is represented in the plot (**E**, below). FasL is expressed on the cell surface (FasL positive, blue line), reaching approximately 61% at 24 h. However, cell death revealed using propidium iodide (PI positive, red line) increased in time, reaching approximately 25% at 48 h.

The kinetics of FasL expression induced by transduction with adenoviruses carrying the *FasL* minigene (Ad-gFasL) and FasL cDNA (Ad-cFasL) were measured in murine hepatocytes (Hepa 1–6) chosen due to the particular high efficiency of transduction. Cells infected with the *FasL* minigene displayed FasL mRNA expression after 8 h, with significantly higher levels after 10 h (Figure 2A, upper panel Ad-gFasL). In variance, the expression of FasL in cells transduced with the adenovirus carrying cDNA was very low with delayed onset at 16 h (Figure 2B, lower panel Ad-cFasL). In both cases, the expression of the Green Fluorescent Protein (GFP) reporter protein preceded the detection of FasL. 

FasL protein expression was assessed in cells transduced with both adenoviruses at incremental viral titers ranging between 0.5 and 10 transfection units (TU) per cell. Western blots performed 40 h after transfection showed a gradual increase in FasL expression in cells transduced with Ad-gFasL (Figure 2C) but not in cells transduced with Ad-cFasL (Figure 2D). Consistent with mRNA detection, GFP proteins were stably detected under all experimental conditions with both adenoviral vectors. 

The timecourse of the FasL expression on the membrane of transduced Hepa 1–6 cells was determined also by flow cytometry (Figure 2E). FasL is expressed on the cell surface (blue line), reaching approximately 61% at 24 h. However, cell death (PI, red line) increased in time, reaching approximately 25% at 48 h. FasL expressed by transduction induced a high level of apoptosis in the transduced Hepa 1–6 cells. Thus, to be able to show the killer potential of the transduced cells, we changed the cell type with one resistant to FasL-induced apoptosis.

**Figure 3:**
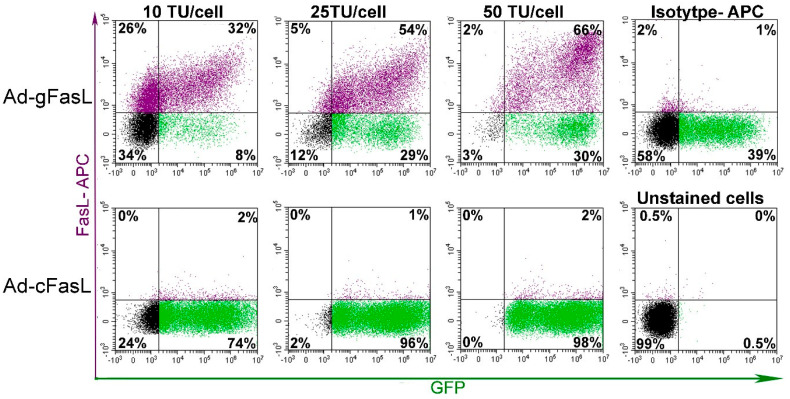
FasL expression in transduced bovine aortic endothelial cells: Cells were transduced with increasing adenoviral titers in the range of 10–50 transduction units (TU) per cell. The cell surface expression of FasL protein was assessed using anti-CD95L antibodies (MFL4 clone) along with intrinsic fluorescence of green fluorescent protein (GFP). Effective induction of FasL expression was observed only for cells transduced with a vector encoding the minigene (Ad-gFasL), whereas only GFP expression was detected following transduction with a vector encoding cDNA (Ad-cFasL).

To evaluate the proper expression of FasL protein on the cell surface, bovine aortic endothelial cells (BAECs) resistant to FasL-induced apoptosis were transduced and monitored for FasL expression using MFL4 antibodies (Figure 3). Cells transduced with Ad-gFasL displayed incremental expression of FasL protein on the surface as a function of transduction units, with robust co-expression of the GFP reporter at high viral titers. Notably, a significant fraction of approximately 30% cells expressed GFP alone, suggesting suboptimal infection considering polarization of FasL and GFP expression alone seen at the lowest adenoviral dose (10 TU/cell). By contrast, cells transduced with Ad-cFasL displayed high levels of GFP expression alone, emphasizing that transduction was effective; however, cDNA carrying adenovirus was not able to induce FasL expression.

### 2.3. Functional FasL Expression in Transduced Cells

**Figure 4:**
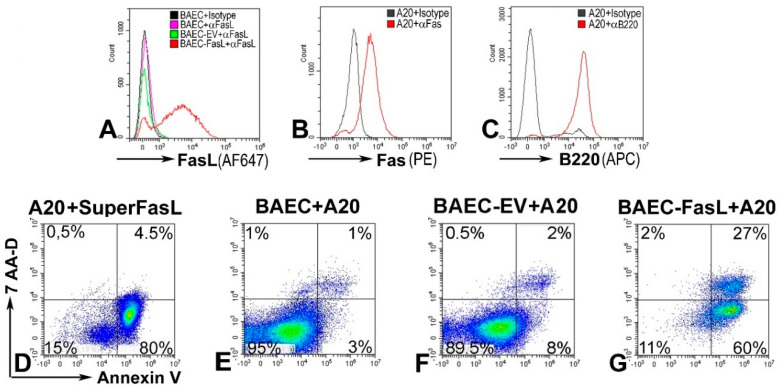
FasL expressed on Ad-gFasL-transduced bovine aortic endothelial cells induces apoptosis of responder cells: (**A**) FasL expression in naïve bovine aortic endothelial cells (BAECs) (pink line), BAECs transduced with an empty virus (EV, green line), and BAECs transduced using Ad-gFasL (red line). (**B**) A20 cells (responder cells) express Fas. (**C**) A20 cells are positive for B220 marker. (**D**) The majority of A20 treated with SuperFasL (trimerized soluble FasL) are apoptotic. (**E**) A20 cells incubated with naïve BAEC are viable. (**F**) BAECs transduced with an empty adenovirus (BAEC-EV) do not induce a significant death of A20 cells. (**G**) By contrast, A20 cells incubated with FasL-expressing BAEC are 87% apoptotic, positive for Annexin V (**F**). In the cell mix, A20 cells were identified based on B220 (**E**–**G**).

Next, we evaluated functionality of the FasL protein expressed on the cell surface. The apoptotic properties of FasL exposed on transduced cells were determined in co-cultures experiments. BAECs transduced with 25 TU/cell showing 60% expression of FasL on the cell surface (Figure 4A) were co-incubated for 24 h with A20 lymphoma leukemia cells, which express Fas (Figure 4B) and, thus, are sensitive to FasL-induced apoptosis (Figure 4D). In co-cultures, A20 were identified based on a B220 lineage marker (Figure 4C). Apoptosis of A20 cells was assessed by incorporation of Annexin-V, and death was determined by 7-AAD. Data showed that 87% of the A20 cells co-incubated with FasL-transduced BAEC (Figure 4F) are apoptotic as compared to 4% Annexin-V-positive A20 cells when they are co-incubated with naïve BAEC (Figure 4E). Altogether, these data demonstrate that FasL is effectively induced and functional in cells transduced with the adenoviral vector encoding the *FasL* minigene.

## 3. Discussion

We described an adapted technology using the AdEasy System for efficient expression of FasL in various cell lines. Following failure of adenoviral vectors encoding the full-length cDNA sequence to induce FasL expression, gene truncation by exclusion of the second intron and part of the distal exon attained good expression of a functional ligand on the cell surface. FasL is encoded by an evolutionarily conserved gene located in chromosome 1, composed of four exons and three introns, with preserved structure and cross-reactivity, both in mice and humans. We recognized that expression of such a large-sized gene in adenoviral vectors might be restricted; therefore, a minigene was constructed to include only part of the noncoding DNA, represented by the first and the third introns, 5′UTR, and a segment of the 3′UTR. The second intron of FasL (4102 bp) was excluded, and the second and the third exons were linked in frame (Figure 1).

Sequence analysis of FasL DNA cloned in the pAdTrack-CMV vector showed no mutation introduced into the exon sequences. Moreover, the two polymorphic sequences of the FasL gene found in BALB/c mice (Thr-184 → Ala-184 and Glu-218 → Gly-218) [21] were present in the cloned *FasL* minigene. Both amino acids are located in the extracellular domain of FasL, and both variants bearing these two substitutions were more active than the sequence described for the *Mus musculus* strain C57BL/6J (NCBI reference sequence: NC_000067.6). The other two mutations found in the introns may be specific for BALB/c mice. The sequence of the minigene was aligned with that from C57BL/6 mice because the sequence of FasL gene from BALB/c mice is not deposited in the GenBank.

Our procedure included several adaptations of the established methodology of adenovirus packaging, amplification, and purification described elsewhere [22,23,24]. The modifications included the following: i) The adenoviral vector was introduced in BJ5193-containing AdEasy1 plasmid by chemical transformation. ii) The selection of recombinant clones was performed by PCR. iii) AD293 cells were transfected using the K2 reagent for viral packaging. iv) One-step ultracentrifugation in a CsCl gradient was employed for viral purification. HEK293 packaging cells for the adenovirus are sensitive to Fas-mediated apoptosis [25], making preparation very difficult. This obstacle was overcome by an apoptosis inhibitor that protects the packaging cells from toxicity inflicted by the FasL adenovirus. Our improvement of the methodology [22,23,24] reduces the cost of the technology, obviating the need for electroporation, and shortens the duration of preparation of the adenoviral vector.

Initial experiments were performed in Hepa 1–6 murine hepatocytes considering that transduction of serotype 5 pAdTrack adenoviruses depends on the Coxsackievirus and Adenovirus receptors (CAR), which are abundantly expressed in the murine liver [26]. Indeed, the murine hepatocytes were transduced with high efficiency and FasL was expressed at high levels (Figure 2). The same high yield of transduction was obtained also on human HepG2 cells (data not shown). Due to the inherent affinity for the liver, this system has been previously used in a study of lipid metabolism, the liver being the major source of plasma apolipoproteins [27,28]. Although under some pathological conditions the liver may be susceptible to apoptosis triggered by the Fas/FasL interaction [26], numerous studies have shown that therapeutic use of FasL in vivo does not cause liver toxicity [29,30]. Human CAR homologs are prevalently expressed in various species, including mice, rats, and pigs [31,32]; therefore, we chose a line of endothelial cells that are inherently resistant to FasL-induced apoptosis [33]. The advantage of cobblestone-shaped cells provides good conditions for cell surface expression of the FasL molecule for co-incubation experiments. Similar results showing FasL functionality were obtained also on a EA.hy926 cell line, but we presented here the results obtained on BAECs since these primary endothelial cells are not modified like those from the EA.hy926 line. Using BAECs, we also demonstrated that the adenovirus can be used to transduce other cell types besides human cells.

Here, we present an effective method of truncation of the FasL gene sequence for insertion in adenoviral vectors. Transient and robust expression of FasL may have multiple potential applications in the induction of transplant tolerance and targeting the apoptotic ligand to various sites of inflammation using molecular and cellular directing moieties.

## 4. Materials and Methods

### 4.1. Cell Culture

AD293 cells (Agilent Technologies, Santa Clara, CA, USA) were grown in DMEM (containing pyruvate, glutamine, and high glucose) supplemented with 10% fetal bovine serum. Hepa 1–6 murine hepatocytes (ATCC, Manassas, VA, USA) and bovine aortic endothelial cells (BAECs) obtained as previously described [34] were grown in DMEM supplemented with 10% fetal bovine serum.

### 4.2. DNA Isolation, Cloning, and Sequencing 

Murine genomic DNA was isolated from the BALB/c lung using a kit from Promega (Madison, WI, USA). FasL cDNA obtained from RNA isolated from mice lung was amplified using the primers FasL170F (KpnI) and FasL 6970R (NotI) (Table S1). DNA detection in agarose gels was achieved by staining with Midori Green (Nippon Genetics Europe, Düren, Germany). The fragment was cleaved using KpnI and NotI restriction enzymes and cloned in the KpnI/NotI restriction sites of the pAdTrack viral vector. Plasmid DNA was purified using the Midi Prep kit from Qiagen (Hilden, Germany).

The cloning strategy for *FasL* minigene is presented in Figure 1. Briefly, a 1–1690 bp FasL gene fragment (*FasL I fragment* containing exon I, intron I, exon II and part of the intron II) was amplified by PCR from murine genomic DNA using primers FasL1F (KpnI) and FasL1690R (SalI), described in Table S1. The FasL gene fragment 4997–6970 (*FasL II fragment* containing part of the intron II, exon III, intron III, and part of the exon IV) was amplified by PCR using FasL 4997F (SalI) and FasL 6970R (NotI) primers (Table S1). The fragments were successively cloned in the pBluescript SK (+) vector (pBSK, Stratagene) in the KpnI/SalI and SalI/NotI sites. The *FasL* minigene was constructed by excision of intron II (both from the 5′ and 3′ends) through overlapping PCR using the FasLovp364R intern and FasLovp409F intern primers described in Table S1. The two PacI sites of the *FasL* minigene (one located in the second intron and the other one located in the 3′UTR the exon IV) were removed. The amplified minigene was directly inserted in the pAdTrack-CMV adenoviral vector (a gift from Bert Vogelstein; Addgene plasmid #16405). At each cloning stage, the obtained plasmids were tested by digestion with the restriction enzymes (Figure S1). Moreover, the ability of the minigene to induce FasL mRNA (proper splicing of the two remaining introns) was checked by transient transfection experiments in HEK293 cells. As shown in Supplemental Figure S1, mRNA of the same size (930 bp) was detected in transfected cells with plasmid encoding the minigene as well as cDNA FasL.

The final construct was sequenced by the Sanger Dye-terminator DNA Sequencing method using the Dye Terminator Cycle Sequencing Kit (Beckman Coulter, Indianapolis, IN, USA) using a Beckman Coulter Sequencer (Beckman Coulter, Indianapolis, IN, USA). Multiple pairs of primers giving overlapped amplification products of approximately 600 bp were designed for sequencing (Table S1).

### 4.3. FasL Adenovirus Packaging and Purification

pAdTrack plasmids containing the *FasL* minigene, FasL cDNA or without any inserted gene (empty vector, EV) were linearized with PmeI and used to transform BJ5183-bacteria containing the pAdEasy1 plasmid (AdEasier-1 cells, a gift from Bert Vogelstein; Addgene plasmid #16399). Competent AdEasier-1 bacteria were prepared using Mix and Go *E. coli* transformation kit (Zymo Research, Irvine, CA, USA). Colonies containing recombinant plasmids were grown on solid Luria-Bertani (LB) medium containing kanamycin and were tested by PCR (Figure S2).

Recombinant DNA was amplified in DH5α competent cells, and 6 μg of the recombinant plasmid was used for the transfection of AD293 cells, using the K2 transfection reagent (kindly provided by Biontex Laboratories GmbH, München, Germany). The adenovirus was further amplified in AD293 cells stably transfected with the plasmid (pLVX-FADD-DD, a gift from Joan Massague, Addgene plasmid #58263) to express a dominant-negative form of FADD [35]. In addition, the packaging cells were cultured in medium containing 0.8 µM apoptosis inhibitor (Caspase-3 inhibitor, R&D System, Minneapolis, MN, USA). The adenovirus was released from the packaging cells by three freezing/thawing cycles. In addition, the virus was precipitated from the culture medium using ammonium sulfate (141 g/500 mL culture medium) and then was resuspended in 10 mM Tris-HCl pH 8 buffer containing 2 mM MgCl_2_. The adenovirus from the cell lysate or resuspended from the precipitate was purified by ultracentrifugation on a discontinuous CsCl gradient (1.2 g/L and 1.4 g/L) at 35,000 rpm in a SW41 Beckman rotor for 18 h at 4 °C.

### 4.4. Titration of Adenoviral Vectors

AD293 cells were seeded at a concentration of 10^5^ cells/well in 12-well plates one day before transduction. The cells were transduced at various dilutions of FasL adenoviral stock ranging between 1/10^4^ and 1/10^7^. The percentage of GFP positive cells was evaluated by flow cytometry after 48 h. Transducing units (TU) per ml were calculated using the formula Titer = F × D × C/V, where F is the frequency of GFP-positive cells (percent of GFP-positive cells/100), D is the viral dilution factor (e.g., 10^5^–10^7^), C is the number of cells at the moment of transduction, and V is the volume of the inoculum.

### 4.5. Viral Transduction for FasL Expression

Murine Hepa 1–6 hepatocytes and bovine aortic endothelial cells were seeded at a density of 9000 cells/cm^2^ and were infected with specified amounts of virus. Two days after transduction, the medium was changed and the cells were processed for further analysis.

### 4.6. RT-PCR

HEK293 transiently transfected using pAdTrack-*FasL* minigene or pAdTrack-FasL cDNA were lysed, and total RNA was isolated using TRIzol (Invitrogen Life Technologies, Carlsbad, CA, USA). After reverse transcription, cDNA was used for amplification by PCR using the FasL1F and FasL 930R primers. Hepa 1–6 cells were transduced with Ad-cFasL or Ad-gFasL for 24 h. Then, at the indicated time, cells were harvested and total RNA was isolated. Corresponding cDNA was obtained by reverstranscription, and the expression was tested by PCR using the FasL 418F and FasL 930R primers (Table S1), which generate products of 512 bp.

### 4.7. Western Blot

Transduced Hepa 1–6 cells were lysed in solubilization buffer and subjected to SDS-PAGE, followed by transfer on nitrocellulose membrane [36]. After blocking, the membrane was incubated with antibodies anti-FasL (MAB5262 R&D Systems, Minneapolis, MN, USA), anti-GFP (#ab290, Abcam, Cambridge, UK), and anti-actin followed by the secondary antibodies labeled with HRP. Chemiluminescent detection was performed using Super Signal West Pico chemiluminescent substrate and was visualized using an ImageQuant LAS-4000 (GE Healthcare Bio-Sciences, Freiburg, Germany).

### 4.8. Flow Cytometric Analysis of FasL Expressed on the Cell Surface

Hepa1–6 cells were transduced with 5 TU/cell Ad-cFasL or Ad-gFasL, and after various periods, FasL expressed on the cell surface was analyzed. BAECs were transduced with 10–50 TU/cell Ad-cFasL or Ad-gFasL, and after 48 h, the cells were harvested and FasL expression was determined. FasL was detected on the surface of the transduced cells with anti-FasL antibodies (MLF4 clone) labeled with Alexa Fluor 647 (#MCA 2896A647 from BioRad, CA, USA), using the CytoFlex (Beckman Coulter, Indianapolis, IN, USA).

### 4.9. Apoptosis Assay in Mixed Cultures

To detect apoptosis induced by FasL, BAECs were transduced with 25 TU/cell for 24 h and thereafter were co-cultured with A20 cells (1:1 ratio) for 24 h. Fas expression was detected on A20 cells using PE-anti CD95 (#152607 BioLegend, San Diego, CA, USA), and B220 was detected using anti B220 (#103260 BioLegend, San Diego, CA, USA) antibody. Cell mixtures were processed for the detection of apoptosis using Annexin V-APC and of cell-death using 7-Amino Actinomycin D (7-AAD), gating on B220^+^ A20 cells.

## 5. Patents

The patent OSIM A/00512 (2020) resulted from a part of the work reported in this manuscript.

## Supplementary Materials

Supplementary materials can be found at https://www.mdpi.com/1422-0067/21/17/6011/s1. Table S1: Primers used for cloning, selection of the recombinants, sequencing, and RT-PCR, Figure S1: *FasL* minigene fragments cloning in pBKS, Figure S2: Selection of recombinant plasmids by PCR.

## Abbreviations

FasL	First apoptosis signal ligand
GFP	Green Fluorescent Protein
FADD	Fas-associated death domain
BAEC	Bovine aortic endothelial cells
