# Supplementary material for: Adenovirus-Mediated FasL Minigene Transfer Endows Transduced Cells with Killer Potential
Source: Int J Mol Sci. 2020 Aug 20;21(17):6011. doi: 10.3390/ijms21176011 (PMC7504687; doi:10.3390/ijms21176011)
Supplement: Supplementary file 1 [file ijms-21-06011-s001.pdf]

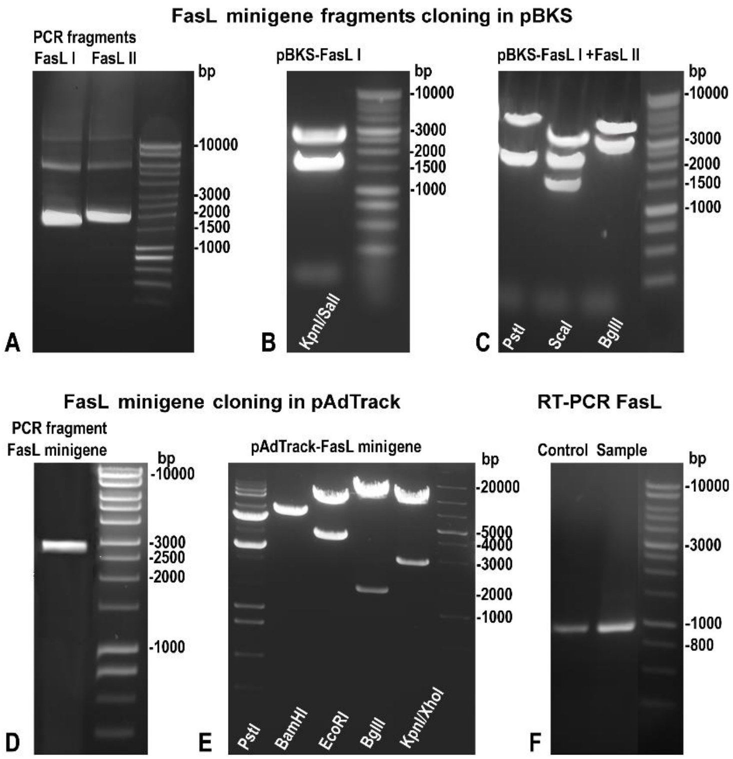

Figure S1. FasL mini-gene construction and testing (A) PCR amplification of FasL gene fragments: FasL I (1707 bp) and FasL II (1992 bp). (B) Digestion of pBKS-FasL I plasmid with Kpn I/Sal I cut out the insert, giving two bands: 1700 bp (FasL I) and 2936 bp. (C) pBKS-FasL I +FasL II digestion: 1959 and 4594 bp for PstI digestion, 1359, 2025, and 3169 bp for ScaI digestion, 2732 and 3821 bp for Bgl II digestion; (D) FasL minigene (2887 bp) obtained by overlapping PCR. (E) pAdTrack-FasL minigene digested with various enzymes: 6090 and 5784 bp (not-resolved on the gel) for BamH I, 3888 and 7986 for EcoRI, 253, 1932 and 9689 for Bgl II, 2887 and 8987 for Kpn I/Xho I. (F) Transient transfection experiments using pAdTrack-FasL minigene in HEK293 cells showed that FasL minigene is functional and produce mRNA in transfected cells (Sample) of the same size (930 bp) as that obtained from the plasmid encoding cDNA FasL (Control).

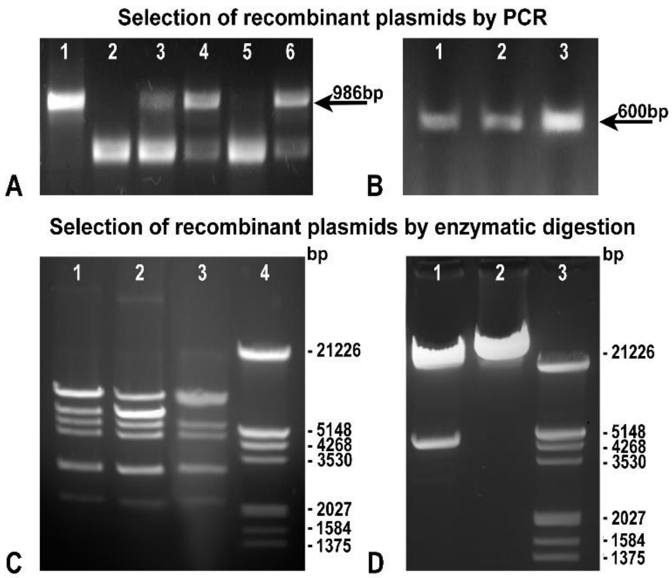

Figure S2. Recombination of pAdTrack-FasL minigene with pAdEasy-1 plasmid. The selection of the recombinant plasmids obtained from pAdTrack-FasL and pAdEasy-1 was performed by PCR for the pAdTrack integrity (A) and the presence of FasL (B). The negative clones for pAdTrack integrity represented potential recombinants (A, lanes 2 and 5) were found to be positive for FasL (B, lanes 1 and 2). When pAdTrack-FasL was used as a template, a band of 986 bp was obtained (A, lane 1) and a band of 600 bp for FasL (B, lane 3). Negative clones for recombination (in which pAdTrack remained intact) are represented in A, lanes 3, 4, and 6. Enzymatic digestion with Hind III of two positive clones (C, lanes 1 and 2) showed a similar pattern with pAdEasy vector (C, lane 3) with additional bands. Pac I digestion of a positive clone cut out a fragment of 4500 bp (D, lane 1). The uncut recombinant plasmid is in D lane 2. DNA ladder is in C, lane 4, and D, lane 3.

Supplemental Table S1. Primers used for cloning, selection of the recombinants, sequencing, and RT-PCR. The cloning primers contain restriction sites for the enzymes (red, italics) named in the right column. Sequencing primers are labeled with *seq*.

Primer	Sequence
FasL1F *(KpnI)*	5′-gg*ggtacc*TGAGGCTTCTCAGCTTCAGATGCAAG
FasL170F *(KpnI)*	5′- ga*ggtacc*CAAGGCTGTGAGAAGGAAACCC
FasL1690R *(SalI)*	5′-ggg*gtcgac*AAGGAGCCAAGGGAGAAGGTCCAGG
FasL 4997F *(SalI)*	5′-ggg*gtcgac*TGGGAATAGACTGTAGGATGATAACC
FasL 6970R *(NotI)*	5′-g*gcggccgc*CCTGGTGCCCATGATAAAGAATAGTAG
FasLovp364R intern	5′-CAGAGGGTGTACTGGGGTTGGCTATAAGCATTTCAAAAGATGATAC
FasLovp409F intern	5′-GTATCATCTTTTGAAAAGCAAATAGCCAACCCCAGTACACCCTCTG
pAdTrack 4631F	5′-CAGTAGTCGGTGCTCGTCCAG
pAdTrack 5616R	5′-TATGGGGGCTGTAATGTTGTCTC
FasL 516Rseq	5′-CAACAACAACAACCCCAAATC
FasL 471Fseq	5′-TGTGGGCTACTGTGTTGCTTGC
FasL 1070Rseq	5′-CCGAGTTAGGGTTTAGCCAC
FasL 965 Fseq	5′-AATTTTGTGTCTTGTTCAG
FasL 1631Rseq	5′-TTACACCAAACCACTTCTTCAC
FasL 1591Fseq	5′-GAGTTCTGGCATTTGTGGGC
FasL 2281Rseq	5′-GGGATGGACCTTGAGTGG
FasL 2143Fseq	5′-TACCTCCTCAGCCACTTTTTC
FasL 418F	5′-AAAGCAAATAGCCAACCCCAG
FasL 930R	5′-CTGGTGCCCATGATAAAGAATAGTAG
FasL 1F	5′-CAAGGCTGTGAGAAGGAAACCC
